# A Commentary On: “NFκB-Activated Astroglial Release of Complement C3 Compromises Neuronal Morphology and Function Associated with Alzheimer’s Disease”. *A cautionary note regarding C3aR*

**DOI:** 10.3389/fimmu.2015.00220

**Published:** 2015-05-07

**Authors:** Trent M. Woodruff, Andrea J. Tenner

**Affiliations:** ^1^School of Biomedical Sciences, The University of Queensland, Brisbane, QLD, Australia; ^2^Department of Molecular Biology and Biochemistry, Institute for Memory Impairment and Neurological Disorders, University of California-Irvine, Irvine, CA, USA

**Keywords:** complement, C3aR, Alzheimer’s disease, SB290157

## Introduction

A recent study by Zheng and colleagues in *Neuron* (1) shows convincing evidence that activation of NF-κB in astrocytes induces expression of the complement component 3 (C3) and impairs neuronal function [summarized by Yates (2)]. The authors also state that the detrimental effects of astrocyte-derived C3 are due to the interaction of the C3 cleavage fragment, C3a, with its receptor (C3aR) on neurons (although no evidence of neuronal C3aR expression, nor generation of the cleavage product C3a, was provided). There are a number of caveats with this latter interpretation due to the use of the so-called C3aR-antagonist SB290157 (3), upon which they base this conclusion. These are summarized below.

## Off-Target Activity

In 2004, Proctor and colleagues (4) reported that when SB290157 was administered to rats (at the same dose as used by Zheng and colleagues), it caused rapid neutropenia. Although SB290157 could block ischemia-induced intestinal pathology in rats, this therapeutic effect was shown not to be due to inhibition of the C3aR, but rather, due to the non-specific effect on circulating leukocyte concentrations (4). This finding was later replicated in mice by an independent group (5). These authors summarized that “*C3aR antagonism does not appear to be responsible for the anti-inflammatory actions of this C3aRA*” (4).

## Agonist Versus Antagonist Activity

In 2005, Mathieu and colleagues (6) reported that SB290157 surprisingly had full agonist (not antagonist) activity at C3aR in many cell systems. They found that SB290157 completely activated the human and mouse C3aR at doses from 30 nM and above. Those authors stated that their results: “*caution against attributing novel roles to C3a based on data obtained with SB290157*.”

Given these studies demonstrating potential off-target and agonist activity, it is difficult to interpret any results obtained when using SB290157 (7, 8). Unfortunately, none of these limitations regarding SB290157 were reported or cited by Zheng and colleagues. There are also other pharmacological considerations regarding the findings by Zheng and colleagues related to the use of this compound, as detailed below.

## Potency

According to the original report by Ames (3), the antagonist potency (IC_50_) of SB290157 at the mouse C3aR is 7 nM. In the *in vitro* assays used by Zheng and colleagues, a 1000 nM dose is used in all experiments. Since dose–response data were not reported, it is unclear whether this much higher concentration was required to observe activity, or whether a lower concentration would still retain functionality. One alternative explanation is that at 1000 nM, SB290157 is having off-target effects or potent agonist activity (4, 6). Dosing SB290157 to C3aR^−/−^ cells (or showing that SB290157 blocks C3a-induced responses in mouse neurons) could help clarify the C3aR-antagonist activity/selectivity of SB290157 in this *in vitro* model.

## Pharmacokinetics

Another consideration regarding SB290157 usage *in vivo* is its pharmacokinetic profile, which has been previously determined in guinea pigs, rats, and mice (3, 4). A 30 mg/kg i.p. dose resulted in peak circulating blood levels of ~7000 ng/mL, with a *t*_1/2_ of ~1.5 h in mice (3). In the study by Zheng and colleagues, a dosing paradigm of 1 mg/kg i.p. three times per week was used. Assuming a linear pharmacokinetic profile, by extrapolation, this would result in peak blood levels of 230 ng/mL, which would drop to below 0.007 ng/mL by 24 h. This equates to a molar concentration in the blood of 437 nM (peak) dropping to below 0.01 nM after just 24 h, and becoming homeopathic within 48 h. Additionally, blood–brain barrier (BBB) penetration data have never been reported for this compound. Even allowing for a generous transfer of drug across the BBB from the circulation, brain levels of this compound would be extremely low (well below the 1000 nM used in the *in vitro* neuronal/glia system). It is hard to reconcile that sufficient C3aR-antagonist could reach the brain with three doses per week to sustain blockade of neuronal C3aR to provide: “*nearly complete rescue of multiple cognitive deficits in APP transgenic mice*” (1). Potentially, other activities of SB290157 as detailed above could be responsible for the *in vivo* efficacy reported in this study.

## Conclusion

In summary, there are several exciting findings reported in the Zheng and colleagues study as highlighted by Yates (2). However, the authors’ conclusion that neuronal dysfunction in Alzheimer’s disease is due to activation of neuronal C3a–C3aR signaling, and that “*C3aR-antagonists may be therapeutically beneficial*,” are premature. Corroborating evidence from studies using C3aR^−/−^ (preferably conditional and inducible) mice crossed with transgenic Alzheimer’s disease models is necessary to support this conclusion.

## Conflict of Interest Statement

The authors declare that the research was conducted in the absence of any commercial or financial relationships that could be construed as a potential conflict of interest.
